# Supplementary material for: Genomic Analysis and Antimicrobial Components of M7, an Aspergillus terreus Strain Derived from the South China Sea
Source: J Fungi (Basel). 2022 Oct 7;8(10):1051. doi: 10.3390/jof8101051 (PMC9605573; doi:10.3390/jof8101051)
Supplement: Supplementary file 1 [file jof-08-01051-s001.zip › jof-1901556 - supplementary.pdf]

Journal of Fungi

MDPI

Supplementary Information

Table S1. NMR data of compound 1 (500 MHz for ^{1}H NMR, 125 MHz for ^{13}C NMR, CD_3OD).

No.	^{13}C (δ)	^{1}H (δ)
5	163.01	
6	66.45	4.20 (2H, d)
7	36.95	2.27(2H, m)
8	14.14	1.03(6H, d)
9	26.12	1.73(2H, m),1.55(2H, m)
10	11.15	1.01(6H, t)

Table S2. NMR data of compound 2 (500 MHz for ^{1}H NMR, 125 MHz for ^{13}C NMR, CD_3OD).

No.	^{13}C (δ)	^{1}H (δ)
2	162.86	
3	67.81	4.11(1H,dd)
5	163.06	
6	66.51	4.21(1H,dd)
7	36.97	2.26(1H,br m)
8	14.13	1.04(3H,d)
9	26.09	1.55(1H,m), 1.75(1H,m),
10	11.11	1.00(3H,t)
11	30.81	2.54(1H,br m)
12	17.80	1.11(3H,d)
13	18.19	1.18(3H,d)

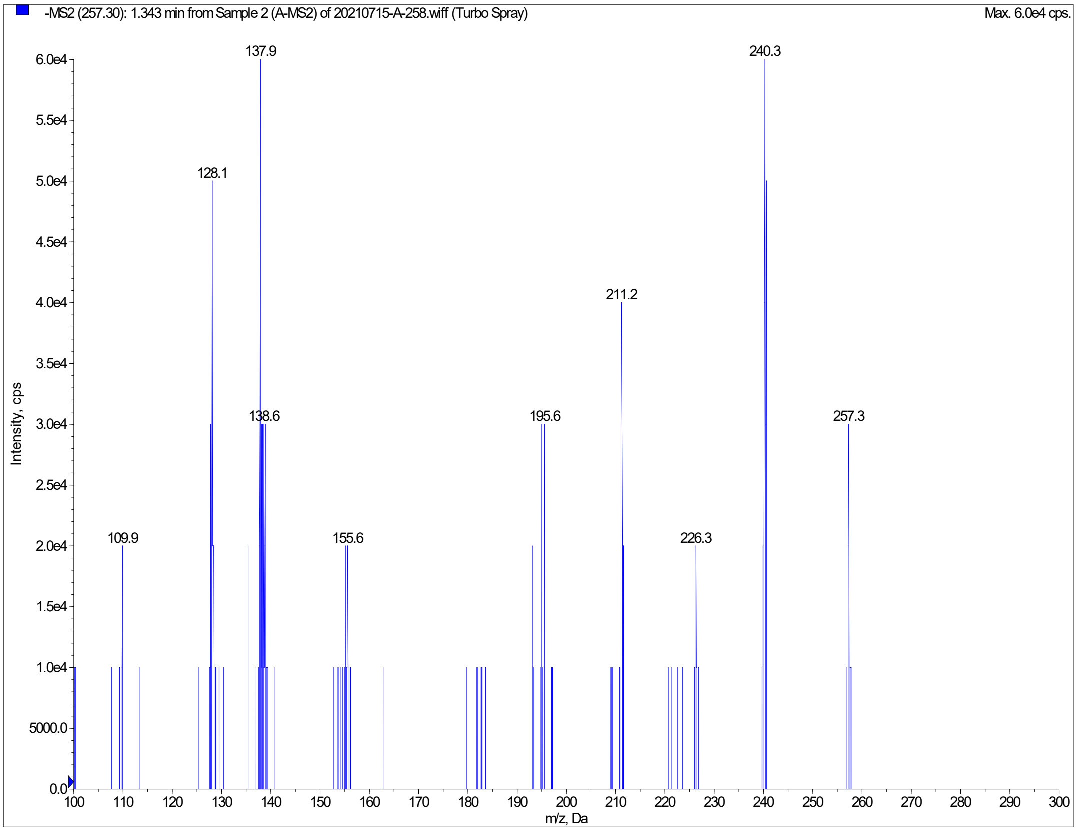

Figure S1. Low resolution mass spectrometry of compound 1.

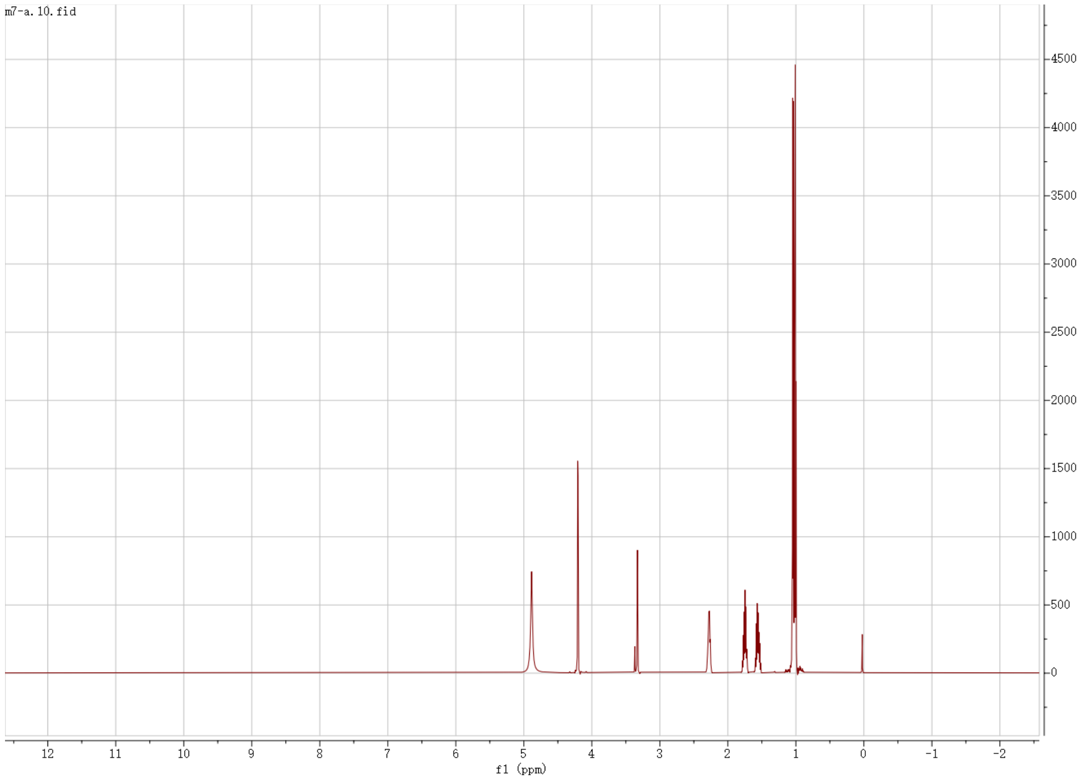

Figure S2. ^{1}H-NMR spectra of compound 1.

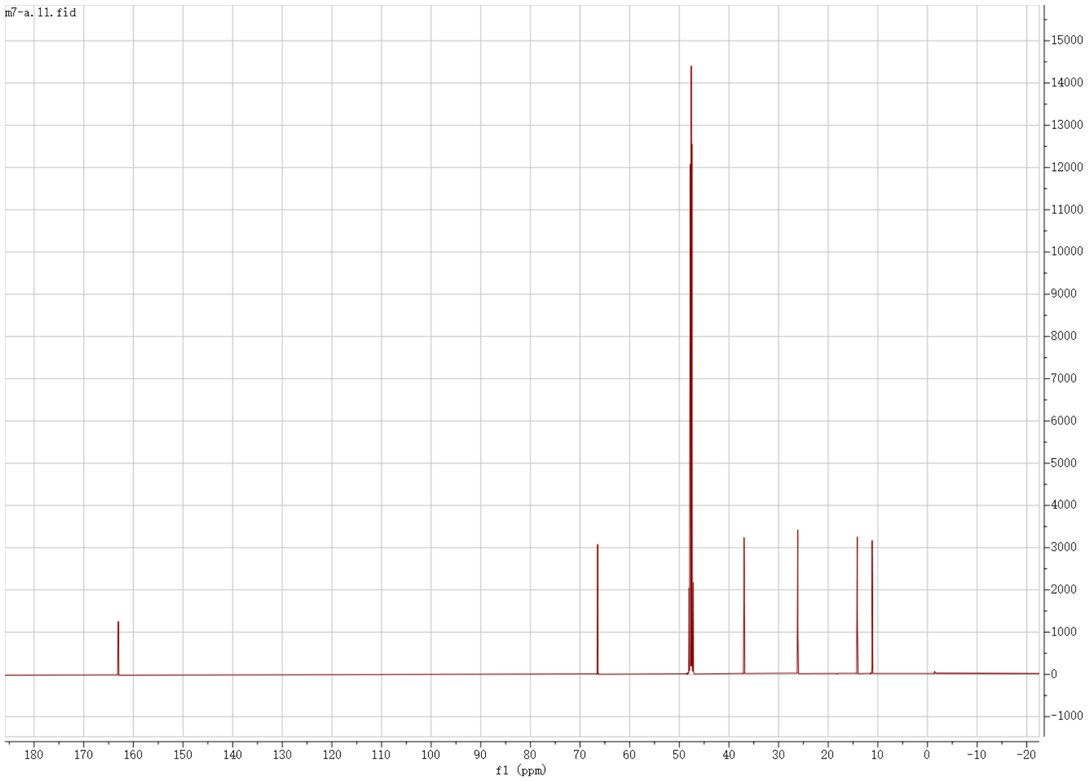

Figure S3. ^{13}C -NMR spectra of compound 1.

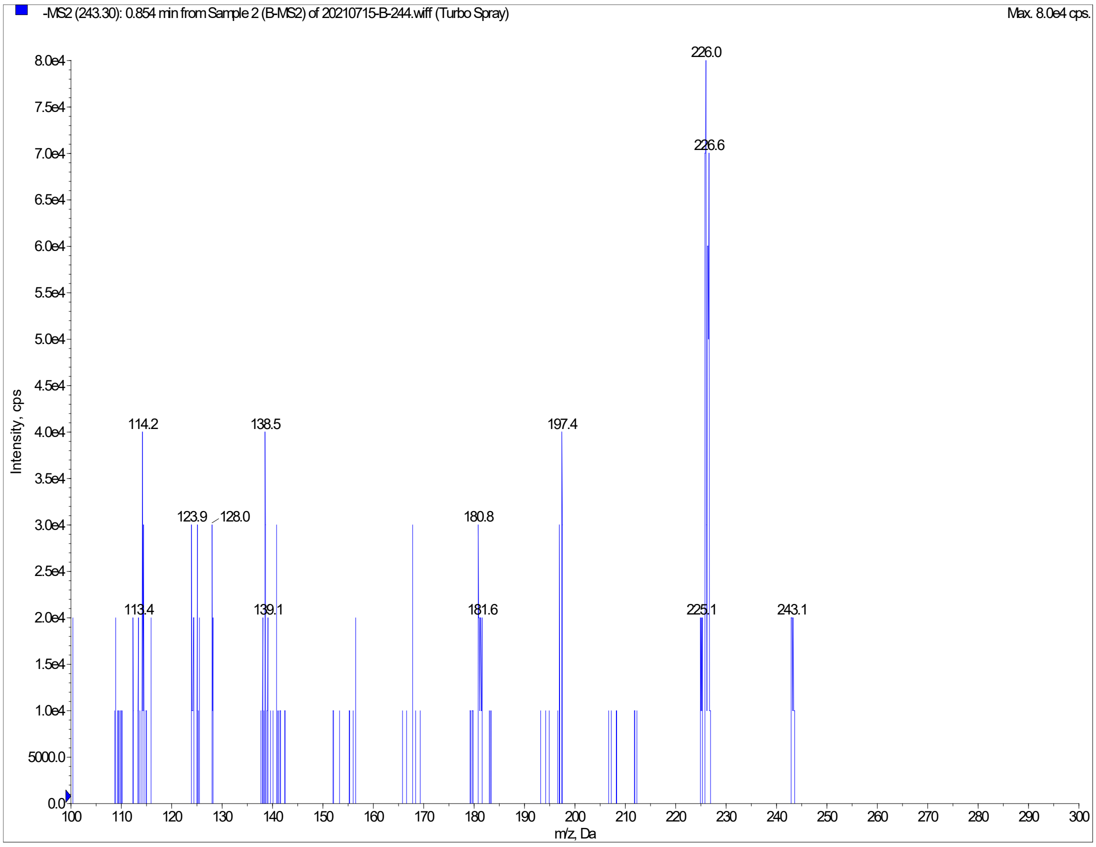

Figure S4. Low resolution mass spectrometry of compound 2.

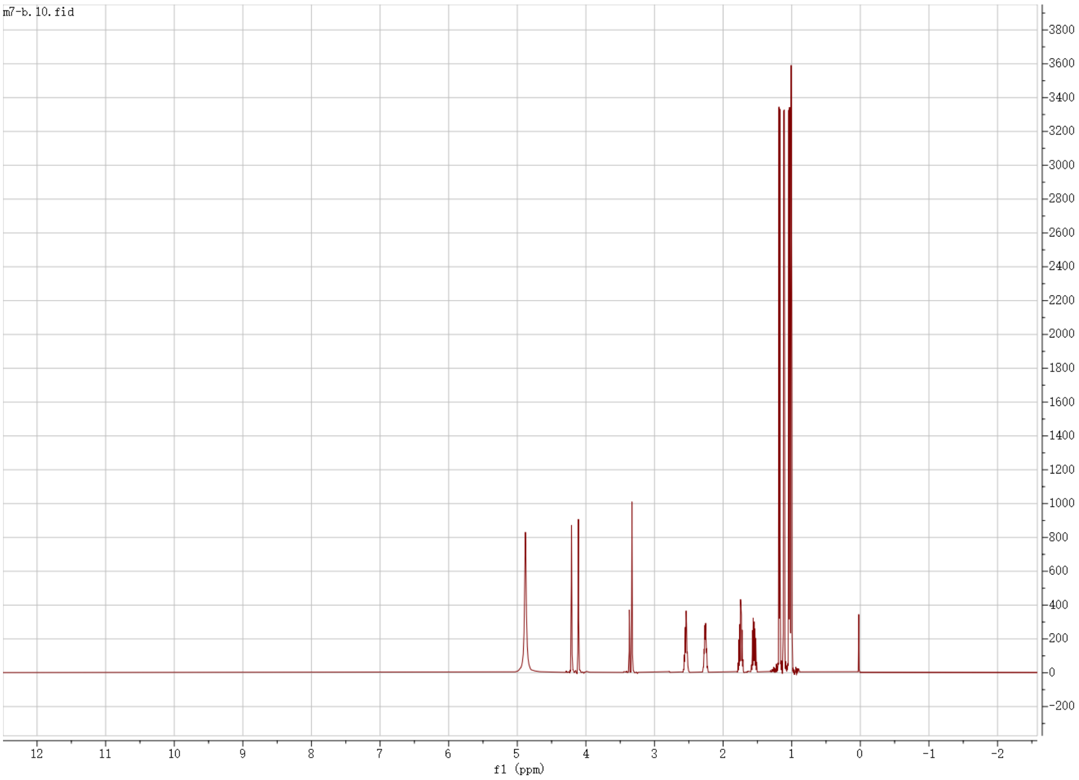

Figure S5. ^{1}H-NMR spectra of compound 2.

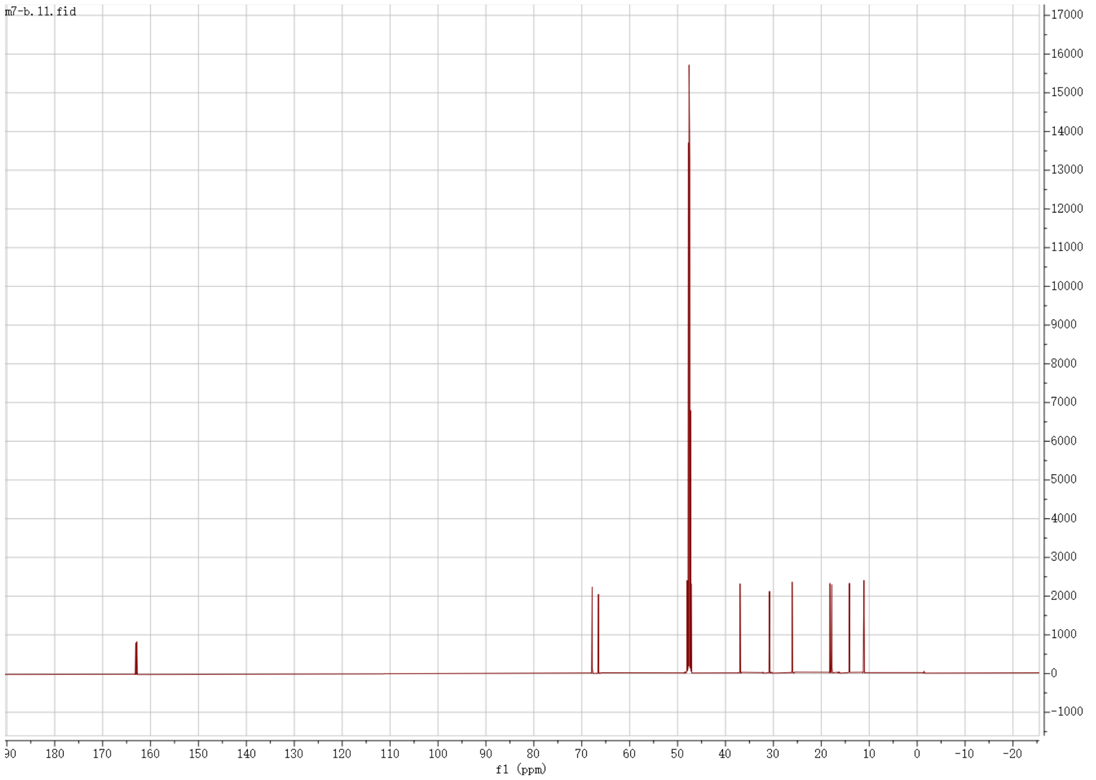

Figure S6. ^{13}C -NMR spectra of compound 2.
